# Computational analysis to investigate the anti-rheumatic potential of plant-based small molecule inhibitor targeting tumor necrosis factor *α*

**DOI:** 10.3389/fphar.2023.1127201

**Published:** 2023-02-07

**Authors:** Sanaya Rehman, Attya Bhatti, Peter John

**Affiliations:** Immunogenetics Laboratory, Department of Healthcare Biotechnology, Atta-Ur-Rahman School of Applied Biosciences, National University of Sciences and Technology (NUST), Islamabad, Pakistan

**Keywords:** rheumatoid arthritis, Dodonaea viscosa, tumor necrosis factor α, small molecule inhibitor, computational analysis, molecular docking, molecular dynamic simulation, drug discovery

## Abstract

**Objective:** This study aimed to assess the anti-rheumatic potential of *Dodonaea viscosa* and to evaluate its bioactive small molecules for their beneficial effects in the management of rheumatoid arthritis.

**Methods:** *In vitro* bioactivity assays were performed to assess the healing potential of *D. viscosa* and statistical analysis was performed by using the linear regression technique. *In silico* analysis was performed to identify the key inhibitors of the disease to target TNF-α. The plant extract was prepared using ethanol solvent *via* the Soxhlet method. Phytochemical and bioactivity testing was performed. Gas chromatography–mass spectrometry (GC-MS) analysis was conducted for bioactive plant compounds. Disease-specific target was shortlisted by HUB gene analysis. Molecular docking and molecular dynamic simulations were run for validation of the results.

**Results:** Phytochemical studies verified the presence of phenols, flavonoids, steroids, sterols, saponins, coumarins, tannins, and terpenoids. The significant antioxidant potential of plant extract was evaluated by the DPPH and Ferric Reducing Antioxidant Power (FRAP) assays, while the anti-inflammatory potential was evaluated by the protein denaturation and Human Red Blood Cell (HRBC) membrane stabilization assays. *In silico* studies revealed that nine of the 480 compounds found in *D. viscosa* (ethanol extract) had drug-like properties. Tumor necrosis factor alpha (TNF-α) was selected as a key disease gene through HUB gene analysis. Results of molecular docking and MD simulation analysis demonstrated that 4-(1-hydroxy-3-oxo-1H-isoindol-2-yl) benzoic acid (PubChemID 18873897), had the best binding affinity with TNF-α amongst all nine compounds.

**Conclusion:** 4-(1-hydroxy-3-oxo-1H-isoindol-2-yl) benzoic acid (PubChemID 18873897), have the potential to be a good small molecule inhibitor of TNF-α against rheumatoid arthritis.

## 1 Introduction

Rheumatoid arthritis (RA) is a chronic autoimmune disease of synovial inflammation and disruption of joints, eventually leading to disability. Although the etiology of the disease is not yet known, studies demonstrate a connection between RA with genetic susceptibility and environmental factors. Other factors that play a major role in the onset of the disease include age, a positive family history, silicate exposure, disease duration, smoking habits, immunological triggers, and psychological factors (Pankowski et al., 2022). RA is one of the highly predominant inflammatory diseases ranging from 0.1% to 2% worldwide adult population. Studies demonstrate an increase in the disease prevalence with increasing age, which affects both genders, whereas the female population is 2–3 times more susceptible to developing the disease (Almutairi et al., 2020).

Tumor necrosis factor alpha (TNF-α) plays a vital role in the prognosis of rheumatoid inflammation. The synovial membrane contains many T-cells that employ cytokines to encourage the growth of osteoclasts, macrophages, and chondrocytes. These cells then induce the synthesis of metalloproteinases and other cytotoxins, which causes the erosion of bone and cartilage (Jang et al., 2021).

Currently, NSAIDs, DMARDs, Steroids, biologics, and Pain-relieving agents are given as a standard course of action against RA. Most of these options have limitations, side effects, or both, which makes targeted approaches against RA’s pathways a much more suitable option. Small molecule inhibitors are considered one of the most efficient ways of targeted therapy and are now widely under research for many diseases. More than 80% of druggable small molecule inhibitors are from natural sources, especially extracted from plant sources. *D. viscosa* is a significant plant with anti-inflammatory properties. (Almarfadi et al., 2022)*.*

**FIGURE 1:**
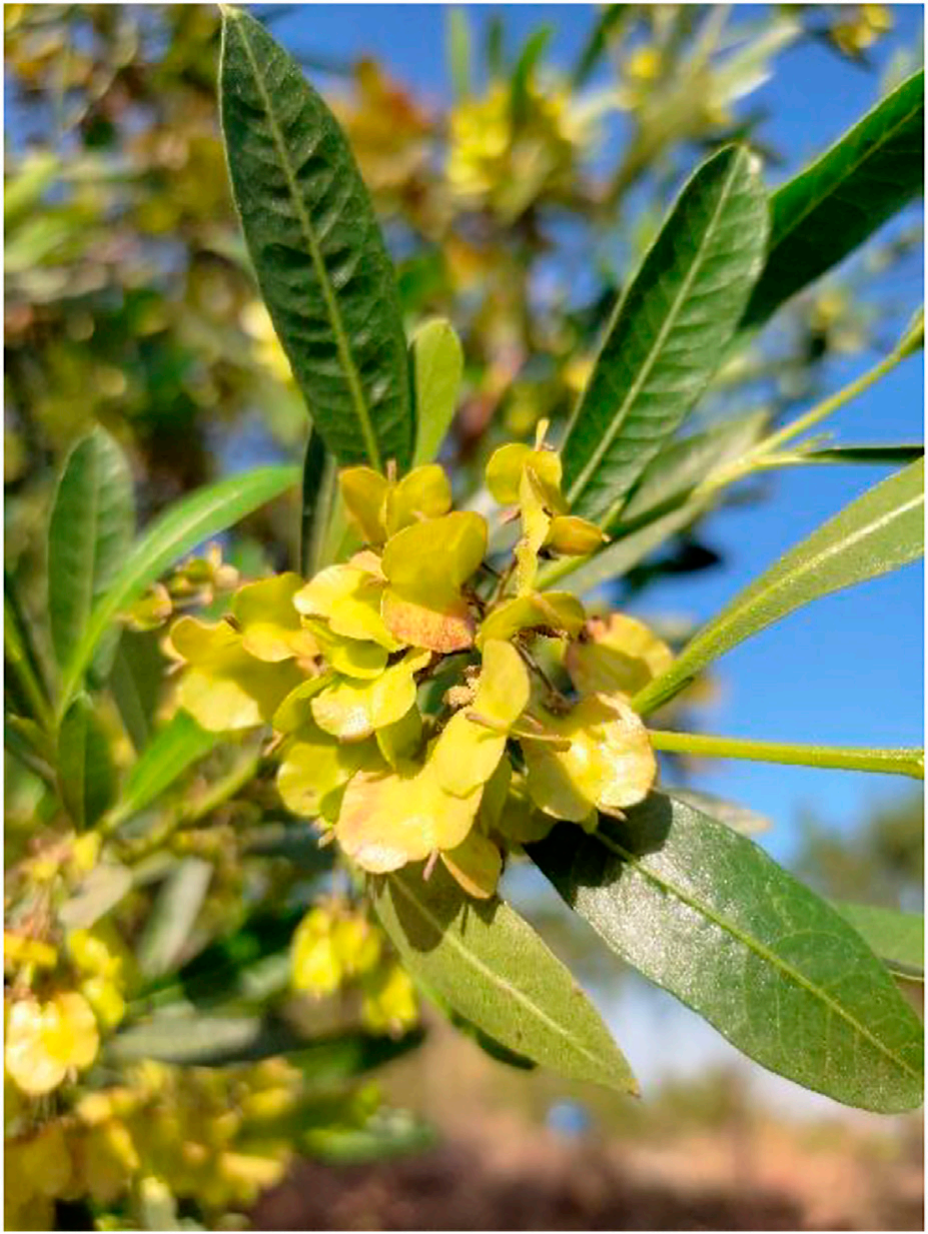
Image of Dodonaea viscosa Linn.

*Dodonaea viscosa* (*L*.) *Jacq*. is a natural species used in traditional medicine that belongs to the Sapindaceae family. It is an evergreen blooming hardy shrub (Figure 1). It is commonly known as called Hop bush in English, whereas, in the Urdu language, it is known as Sanatha (Mokbel, 2020). It has anti-inflammatory and analgesic capabilities due to its active chemical ingredients. Antioxidant (Ramkumar and Periyasamy, 2022) and wound-healing properties (Nayeem et al., 2021) have also been reported in studies, which may aid in the formulation of pharmaceutical medications (Beshah et al., 2020).

*D. viscosa* has remarkable medicinal and therapeutic properties including both traditional and modern applications. It has been used as a folk remedy against many health disorders even for rheumatoid arthritis and joint pain. Due to the presence of various therapeutic properties (Siraj Khan et al., 2022), various parts of the plant are used as antiviral, analgesic, anti-inflammatory, anti-microbial, anti-ulcerogenic, and laxatives (Malik et al., 2022).

Therefore, this study aimed to evaluate the antioxidant and anti-inflammatory potential of *D. viscosa* and to find potential TNF-inhibitor(s) from the bioactive compounds of *D. viscosa* using computational tools, which may be used to treat RA.

## 2 Materials and methods

### 2.1 *In vitro* evaluation of therapeutic bioactive compounds in *D. viscosa*

#### 2.1.1 Plant collection

*D. viscosa* was collected from Margalla Hills, Islamabad, Pakistan. Voucher specimens containing plant leaves, flowers, roots, and bark were deposited in the herbarium. After verification and authentication of the plant, accession number (00046453) was allotted by the Pakistan Museum of Natural History (PMNH), botanical sciences division, Shakarparian, Islamabad, Pakistan.

#### 2.1.2 Plant extract preparation

The leaves of the plant were separated, cleaned, and washed under running tap water to remove dust. Then, they were shade dried at room temperature, for a week. The dried leaves were crushed and ground to a fine powder with the help of a mechanical blender. Later, the powder was stored in an air-tight container with proper labeling for further usage.

Extracts were prepared according to (Luque de Castro and García-Ayuso, 1998) protocol by the soxhlet extraction method with slight modifications. For extraction, 15 g of the dried plant was dissolved in 200 mL of ethanol. Extraction was conducted at the required 50°C–60°C for 2–3 days. The extract was filtered, dried at room temperature, and stored at 4°C in the refrigerator for further experimentation.

#### 2.1.3 Phytochemical screening of *D. viscosa*

The phytochemical analysis of leaf extract was performed using standard methods (Hossain, 2019).

#### 2.1.4 Antioxidant determination

DPPH (2,2-diphenyl-1-picryl-hydrazyl-hydrate) assay: The reported method (Ho et al., 2001) determines the free radical scavenging capacity. According to the key principle of this assay, free radicals form a stable complex at room temperature in the presence of an antioxidant molecule. DPPH, which is originally violet-colored turns into a colorless solution once the stable complex is formed.

Requirements: DPPH, methanol, ascorbic acid, plant extract.

Firstly, 1 mg of DPPH bought from Sigma–Aldrich was dissolved in 25 mL of methanol. The tube was wrapped and then incubated at 4°C for 30 min before use. An extract of *D. viscosa* was prepared by dissolving 1 mg in 1 mL of methanol. In a 96-well plate, dilutions of various concentrations (10, 20, 30, 40, 50, and 60 μg/mL) were added in triplicates with a total volume of 200 µL in each well. Ascorbic acid was used as a standard drug. The plate was wrapped and incubated in dark for about 1 hour. Absorption (OD) was taken, and percentage inhibition was calculated from the given formula.

$$\%\;inhibition=\left(1-Absorbance\;of\;sample/\;Absorbance\;of\;control\right)\times 100$$

Ferric Reducing Antioxidant Power (FRAP) assay: The reported assay is an adaptable and economical tool for determining the antioxidant potential of the sample. According to the FRAP assay principle, the ferric-tripyridyltriazine (Fe+3 -TPTZ) complex is reduced to ferrous-tripyridyltriazine (Fe+2 -TPTZ) by the antioxidants of a sample at low pH. The standard protocol from (Benzie and Devaki, 2017) was used with slight modifications.

Requirements: 1% KFeCN, 10% TCA, 0.1% FeCl_3_, sodium phosphate buffer 0.2 M maintained at 6.6 pH, ascorbic acid, sample solution.

First, dilutions of plant extract/standard were made with varying concentrations (25, 50, 75, 100, 125, 150, and 175 μg/mL) in falcon tubes. Each tube contained 1 mL sample dilution, 2.5 mL sodium phosphate buffer, and 2.5 mL of 1% KFeCN. The tubes were then incubated for 20 mins at 50°C. After that, 2.5 mL of 10% TCA was added and mixed well. Next, the mixture was centrifuged at 3000 rpm for 10 min. After centrifugation, 2.5 mL of supernatant was obtained and added to a new falcon tube where 2.5 mL of FeCl_3_ was added. Absorbance was taken at 700 nm in a spectrophotometer, and the results were analyzed. Ascorbic acid was used as a standard.

#### 2.1.5 Anti-inflammatory determination

Protein denaturation assay: Denaturation of proteins is caused by inflammation in which the structure of the protein is lost due to many external factors such as heat. The protocol obtained from (Umme and Hasan, 2019) was used with slight modifications. The principle of this assay is to evaluate the anti-inflammatory activity of plant extract where it effectively inhibits protein denaturation.

Requirements: 1% BSA aqueous solution, PBS 0.2 M, diclofenac potassium, plant extract.

Firstly, different plant extract concentrations (100, 200, 300, and 400 μg/mL) were prepared. Then 0.2 mL of 1% BSA solution and 2.8 mL of PBS (0.2 M) were added to the same reaction tubes and heated in a water bath at 37°C for 15 mins. They were then placed in an incubator at 70°C for 5 mins. Tubes were allowed to cool at room temperature for a few minutes. Finally, absorbance was taken at 620 nm. Diclofenac potassium was used as a standard drug. Percentage inhibition was calculated from the given formula.

$$\%\;inhibition=\left(1-Absorbance\;of\;sample/\;Absorbance\;of\;control\right)\times 100$$

HRBC membrane stabilization assay: Human red blood cell membrane stabilization assay is performed to estimate the anti-inflammatory potential of our sample *in vitro*. A standard method from (Parvin et al., 2015) was followed with slight modifications.

Requirements: 0.85% w/v normal saline, phosphate buffer solution (7.4 pH), whole blood, diclofenac potassium, plant extract.

First, 5 mL of whole blood was drawn from a healthy volunteer. It was then centrifuged at 3,000 rpm for 5 min. Then, washing with (0.85% w/v) normal saline was done thrice. The remaining blood volume was then measured, and 1 mL of that blood was taken for making 10% (v/v) blood suspension with a normal saline solution. After that, various dilutions of different concentrations (100, 200, 300, and 400 μg/mL) were made with extract/standard. In another falcon tube, 0.5 mL of prepared sample dilution was added. Then 1 mL of normal saline, 0.5 of l blood suspension, and 1 mL of phosphate buffer solution (7.4 pH) were added in falcon, respectively. These tubes were incubated at 37°C for 30 min in a shaking water bath. After that, the tubes were centrifuged at 2,500 rpm for 5 mins, and absorbance was taken at 540 nm. Phosphate buffer solution (PBS) was used as control whereas, diclofenac potassium was used as a standard drug.

$$\%\;inhibition=\left(1-Absorbance\;of\;sample/\;Absorbance\;of\;control\right)\times 100$$

#### 2.1.6 Statistical analysis

The correlation of the data was analyzed statistically using the linear regression technique. This analysis was carried out using the OriginPro 2022 software.

### 2.2 In-silico evaluation of phytochemicals in *D. viscosa* and its potential targets against the treatment of rheumatoid arthritis

#### 2.2.1 GC-MS and collection of data

Data on plant phytochemicals were obtained by the Gas chromatography-mass spectrometry (GC-MS) technique of ethanol plant extract. For this, 1 mg of dried plant extract was dissolved in 1 mL of ethanol solvent and subjected to GC-MS analysis in QP-2020 SHIMADZU (Japan) system interfaced to SH-Rxi-5Sil mass spectrophotometer available in USPCASE, NUST. A standard procedure was used to generate a library of polar compounds and then a further *in silico* study was conducted.

#### 2.2.2 Screening lead compounds based on drug-likeliness

After retrieving phytochemicals data of *D. viscosa* from the GCMS results, 480 compounds were screened by various software based on certain criteria as mentioned in Table 1.

**TABLE 1:** Inclusion/exclusion criteria for shortlisting of the compounds.

	Name	Criteria
1	Lipinski and other violations	Zero violations
2	Molecular weight (MW)	150–500
3	TPSA	20–130
4	GI absorption	High
5	BBB permeability	No
6	Medicinal chemistry violations	Zero violations
7	Water solubility	Soluble/very soluble
8	Cardiotoxicity (hERG prediction)	No
9	CYP450 inhibition	Non-inhibitor

The SwissADME online web-based tool on different parameters including molecular weight, BBB permeability, Lipinski rule, GI absorption, and TPSA. Pred-hERG 4.2 (http://predherg.labmol.com.br/), a free web computational tool was used for predicting cardiac toxicity since lethal cardiac arrhythmia is caused by the blockage of the hERG (K+) channels and therefore, plays a key role in drug development. OCHEM (https://ochem.eu/home/show.do), an online chemical database with a modeling environment was used for analyzing CYP450 inhibition. CYP450 enzymes are membrane-bound hemoprotein that plays a significant role in the metabolism of drugs and xenobiotics and maintains hemostasis. Moreover, drug-drug interactions are triggered by the induction or inhibition of these enzymes. After applying all these parameters, the final shortlisted compounds had the best biocompatibility and drug-likeliness.

#### 2.2.3 Computational RA target prediction and network construction

Databases like Comparative Toxicogenomic Database (CTD) and Therapeutic Target Database (TTD) were used to obtain a list of the top two hundred potential biological targets of RA. An excel file was generated for all biologically active RA targets. These targets were then used to construct a protein-protein network on STRING software.

#### 2.2.4 HUB gene analysis

Cytoscape 3.9.1 software from https://cytoscape.org/ with its extensions like Cytohubba and STRING was installed. The STRING network of 200 RA genes was exported to Cytoscape 3.9.1. Cytohubba was launched to calculate the node score of the top disease gene from the network which in this case was TNF-α.

#### 2.2.5 Structure retrieval

Structures of shortlisted compounds were obtained from PubChem and downloaded in SDF format. Whereas protein structures were retrieved from Protein Data Bank (PDB) from https://www.rcsb.org/.

#### 2.2.6 Preparation of structures for molecular docking

After structure retrieval from PDB, the protein structure was prepared for molecular docking by removing water molecules, ions, and ligands. Whereas ligand structure was similarly prepared by AutoDock software. Then, the binding sites of the protein were identified by reviewing the literature and validating by using PyMOL and Discovery studio.

#### 2.2.7 *In silico* molecular docking and MD simulation

PyRx software was used for the docking of the prepared structures. The docking results displayed nine conformations with their binding energy scores and RMSD values. The docked structures were visualized in PyMOL and Discovery studio to analyze their binding amino acids and type of interactions. The results were confirmed by redocking the known protein complex from PDB.

Among all nine conformations generated by PyRx software, the complexes with the best binding energies were used for further MD simulation analysis since the lowest binding energy means they are the most stable complexes.

Molecular dynamic simulation analysis was performed using GROMACS where a controlled three-dimensional environment was created for the protein-ligand complex with monitored parameters such as temperature, pressure, and ions. MD simulation was run for 100 ns, the complexes were visualized using VMD software for the changes in their binding affinity.

## 3 Results

### 3.1 Phytochemical testing

The results of phytochemical testing (Table 2) demonstrate that the leaves of *D. viscosa* contain alkaloids, phenols, flavonoids, tannins, coumarins, terpenoids, sterols, saponins, and cardiac glycosides whereas, anthraquinones, anthocyanins, and glycosides are absent.

**TABLE 2:** Phytochemical testing results of Dodonaea viscosa extract.

S. No.	Test	*D. viscosa*	Appearance
1	Alkaloid	+	Yellow precipitate
2	Phenols	++	Bluish black color
3	Anthraquinones	-	Violate or red color
4	Flavonoids	+	Yellow precipitate
5	Anthocyanins	-	Red to Bluish color
6	Tannins	+	Greenish to black color
7	Coumarins	++	Yellow color
8	Terpenoids	++	Deep red color
9	Sterols	++	The red color appears at the lower layer
10	Saponins	+	Froth appearance
11	Glycosides	-	Blue to Green color
12	Cardiac glycosides	+	Violate color below the Brown color

### 3.2 Total phenolic content (TPC) and total flavonoid content (TFC)

The quantitative assessment of total phenolic content is expressed as mg gallic acid equivalent per Gram of dry extract i.e., mg GAE/g dry extract. The results demonstrated the presence of significant amounts of phenols in the ethanol extract of *D. viscosa*; 494.44 ± 0.004 mg GAE/g dry extract. TFC is expressed as mg rutin equivalent per Gram of dry extract i.e., mg RE/g dry extract. The results indicated the presence of significant amounts of flavonoids in the ethanol extract of *D. viscosa*; 883 ± 0.003 mg RE/g dry extract.

### 3.3 Antioxidant assays

#### 3.3.1 DPPH assay

The results of DPPH (2, 2-diphenyl-1-picrylhydrazyl) demonstrate the antioxidant activity of *D. viscosa*. They were evaluated by using the standard formula:

$$\%\;inhibition=\left(1-Absorbance\;of\;sample/\;Absorbance\;of\;control\right)\times 100$$

**FIGURE 2:**
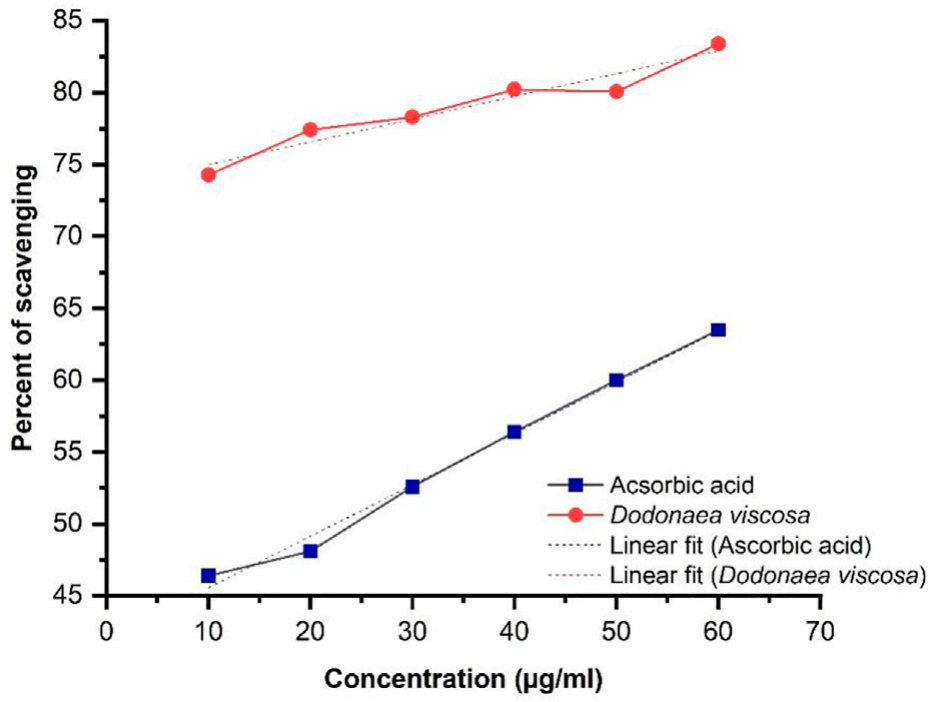
DPPH activity of ethanol extract of Dodonaea viscosa leaves.

Linear regression illustrated in Figure 2, shows an increase in % inhibition with increasing concentration of extract. Ascorbic acid was taken as a positive control. The ethanolic plant extract has significantly higher antioxidant activity i.e., *p-value ≤0.05 as compared to ascorbic acid. Statistical analysis was performed using Origin software.

DV (ethanol extract); *R* ^2^ = 0.930, *p*-value 0.0018.

#### 3.3.2 FRAP assay

**FIGURE 3:**
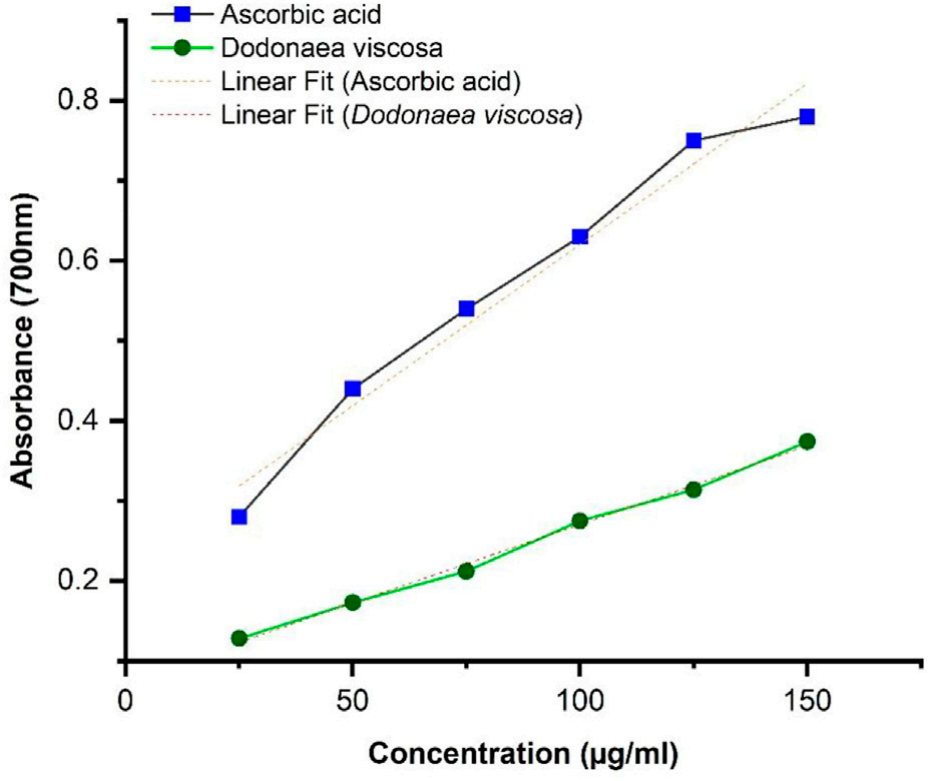
FRAP activity of Dodonaea viscosa leaves.

The results of FRAP were calculated by taking absorption at 700 nm. The results were obtained by linear regression illustrated in Figure 3, showing that the antioxidant potential of ethanolic plant extract is concentration dependent. It can be seen that *D. viscosa* possesses a good antioxidant potential with a *p*-value ≤0.0001, whereas compared to ascorbic acid, the absorbance is slightly lower. Statistical analysis was performed using Origin software.

DV (ethanolic extract); *R* ^2^ = 0.995, *p*-value ≤0.0001.

### 3.4 Anti-inflammatory assays

#### 3.4.1 Protein denaturation assay

The protein denaturation assay demonstrates the anti-inflammatory potential of Dodonaea viscosa. The results were evaluated by using the following formula:

$$\%\;inhibition=\left(1-Absorbance\;of\;sample/\;Absorbance\;of\;control\right)\times 100$$

**FIGURE 4:**
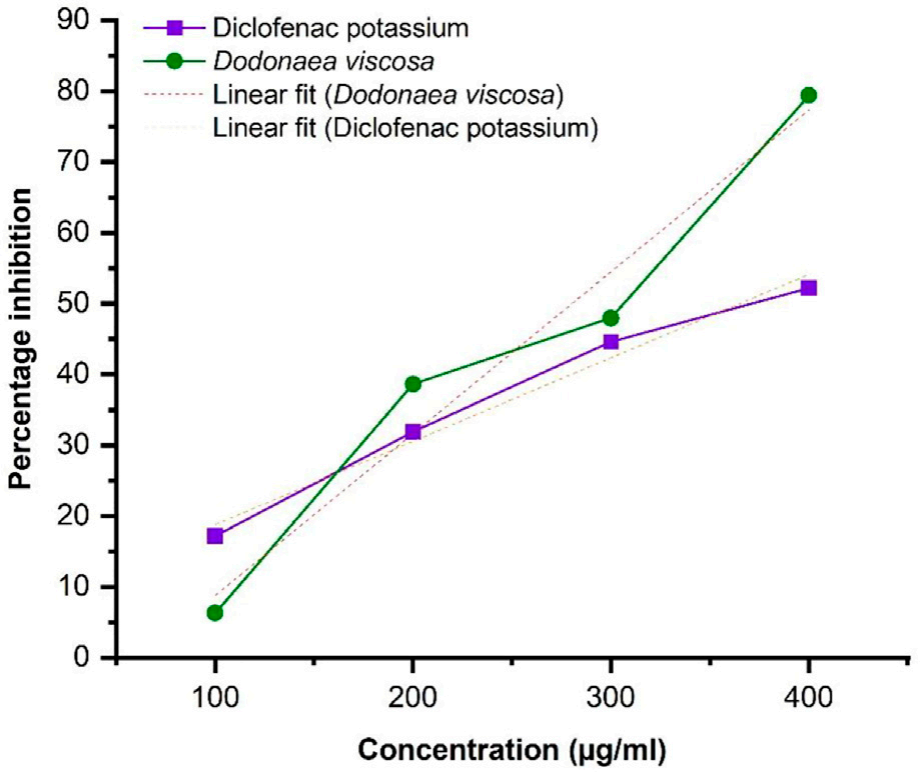
Protein denaturation activity of Dodonaea viscosa leaves.

Linear regression illustrated in Figure 4, shows an increase in % inhibition with increasing concentration of extract. Diclofenac potassium was taken as a positive control. The ethanol plant extract has significantly higher anti-inflammatory activity i.e., *p-value ≤0.05 with respect to the standard. Statistical analysis was performed using Origin software.

DV (ethanol extract); *R* ^2^ = 0.9624, *p*-value 0.01897.

#### 3.4.2 HRBC assay

The HRBC membrane stabilization assay results demonstrate the anti-inflammatory potential of Dodonaea viscosa. The results were evaluated by using the standard formula:

$$\%\;inhibition=\left(1-Absorbance\;of\;sample/\;Absorbance\;of\;control\right)\times 100$$

**FIGURE 5:**
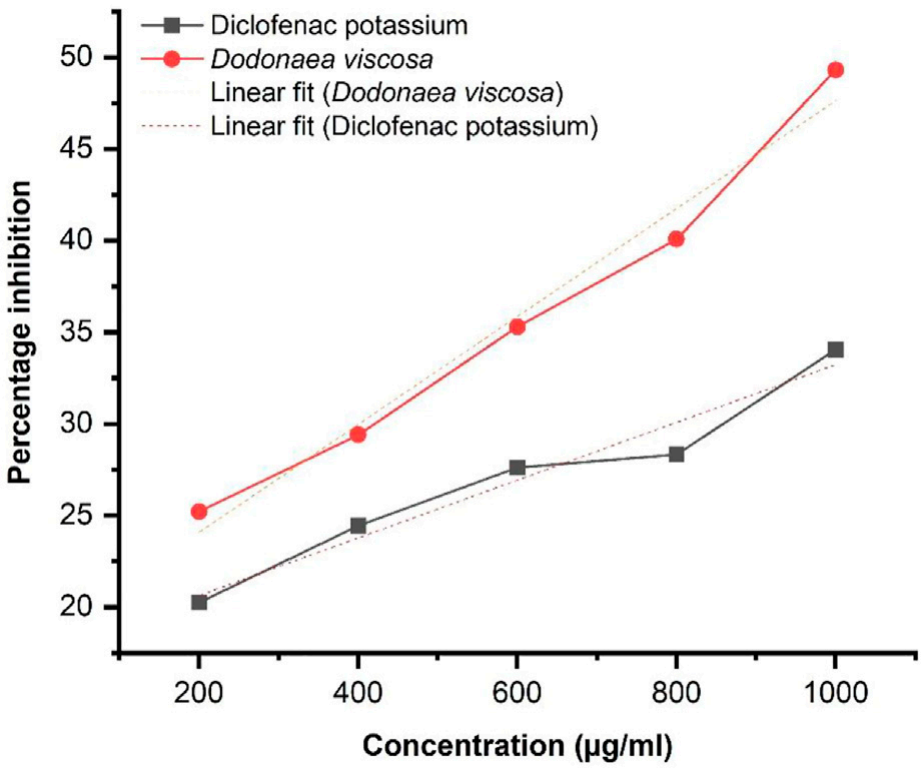
Percent inhibition of HRBC membrane stabilization of ethanolic *D. viscosa* extract.

The graphical result obtained by linear regression as illustrated in Figure 5, shows an increase in % inhibition with increasing concentration of extract at 620 nm. Diclofenac potassium was taken as a positive control The ethanol plant extract has significantly higher anti-inflammatory activity i.e., *p-value ≤0.05 with respect to the standard. Statistical analysis was performed by using Origin software.

DV (ethanolic extract); *R* ^2^ = 0.9788, *p*-value 0.00132.

### 3.5 *In silico* analysis of TNF-α with its inhibitors

The results of GC-MS gave a list of 480 bioactive plant compounds which were screened by applying various parameters like Lipinski’s rule of five, molecular weight, GI absorption, Blood-brain barrier (BBB) permeability, water solubility and cardiotoxicity from SwissADME, Pred-hERG, and OCHEM for determining the compounds with the best biocompatibility. After shortlisting, nine final compounds obtained were as follows:

(1) 7-(2-Fluorophenyl)-4H,7H-[1,2,4]triazolo[1,5-a]pyrimidine-5-carboxylic acid
(2) 4-Piperidinecarboxamide, 1-[2-(3,5-dimethyl-1H-pyrazol-1-yl)acetyl]-
(3) 2-(4-Hydroxypyrimidin-2-ylsulfanyl)-N-(5-methylisoxazol-3-yl)acetamide
(4) Acetamide, N-methyl-2-[(5-methyl-1,3,4-thiadiazol-2-yl)thio]-N-phenyl
(5) 5-(P-acetylaminophenylsulfonyl)dihydro-1,3,5-dioxazine
(6) 4-(1-Hydroxy-3-oxo-1H-isoindol-2-yl)benzoic acid
(7) 3-(2,3-Dihydro-1,4-benzodioxin-6-yl)-3-hydroxy-2H-isoindol-1-one
(8) 4-Acetyl-5-(furan-2-yl)-3-hydroxy-1-(pyridin-3-yl)-5H-pyrrol-2-one
(9) 5,6,7-Trimethyl-[1,2,4]triazolo[1,5-a]pyrimidin-2-ylsulfanyl)-acetic acid.

The disease target, TNF-α (PDB: 2az5) was selected for molecular docking through the method of HUB gene analysis. The selected PDB 2az5 structure had a resolution of 2.1Å, indicating good structural quality.

All the shortlisted nine compounds with good biocompatibility were docked with TNF-α (2az5), and the compound with the lowest binding energy was selected since the complexes with the lowest binding energies have the most stable conformation (Table 3). As shown in Table 3, a compound named, 4-(1-hydroxy-3-oxo-1H-isoindol-2-yl) benzoic acid (PubChemID 18873897) having the lowest binding energy was selected and docked with TNF. The docking result of TNF with plant compound was validated by re-docking TNF with its original ligand (PDB:2az5).

**TABLE 3:** Molecular docking results for ligands bound to TNF-α receptor (2AZ5).

Ligand	PubChem ID	Binding energy (kcal/mol)
**2AZ5 Inhibitor**		−8.6
Compound 1	135645702	−8
Compound 2	951128	−6.9
Compound 3	753333	−6.7
Compound 4	555687	−6.6
Compound 5	536713	−6.9
**Compound 6**	**18873897**	**−8.2**
Compound 7	91723927	−8
Compound 8	91710979	−7
Compound 9	672041	−6.6

The bold values indicate the lowest binding energy among all the docked complexes.

**FIGURE 6:**
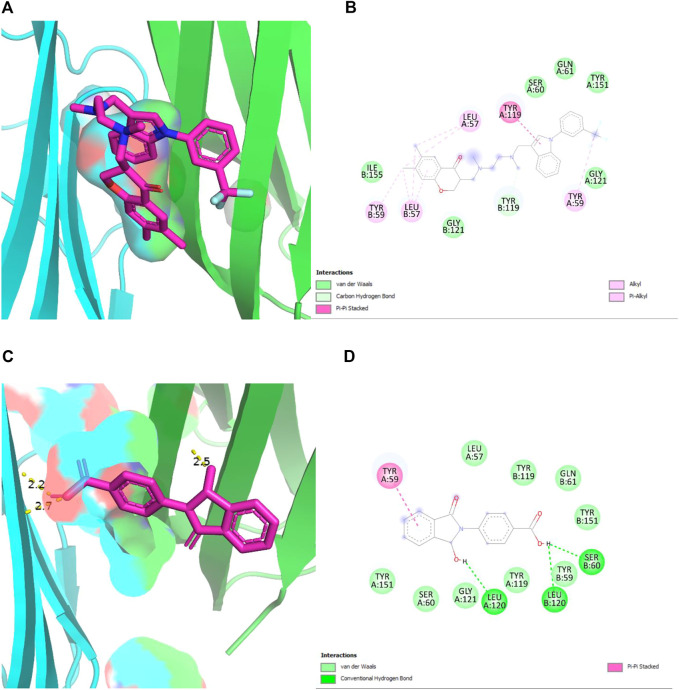
**(A,B)** 2D and 3D structures of TNF-α (PDB 2AZ5) docked with its standard inhibitor. The interaction shows a strong bonding due to the presence of many Van der Waal interactions, with an overall binding energy of −8.6 kcal/mol. **(C,D)** 2D and 3D structures of TNF-α (PDB 2AZ5) docked with plant compound, show the lowest binding energy of −8.2 kcal/mol. The presence of three hydrogen bonds provides stability to the complex.

In Figures 6A, B, the original inhibitor is docked in the pocket of TNF, where Tyr 59 plays an essential role in providing strong binding of the complex along with many other Van der Waal interactions. The overall binding energy is −8.6 kcal/mol with no hydrogen bonds. In Figures 6C, D, plant compound, 4-(1-hydroxy-3-oxo-1H-isoindol-2-yl) benzoic acid (PubChemID 18873897) is docked with TNF in the same pocket. Three hydrogen bonds are formed in this interaction at LeuA:120, LeuB:120, and SerB:60. The overall binding energy is −8.2 kcal/mol with a stable conformation.

**FIGURE 7:**
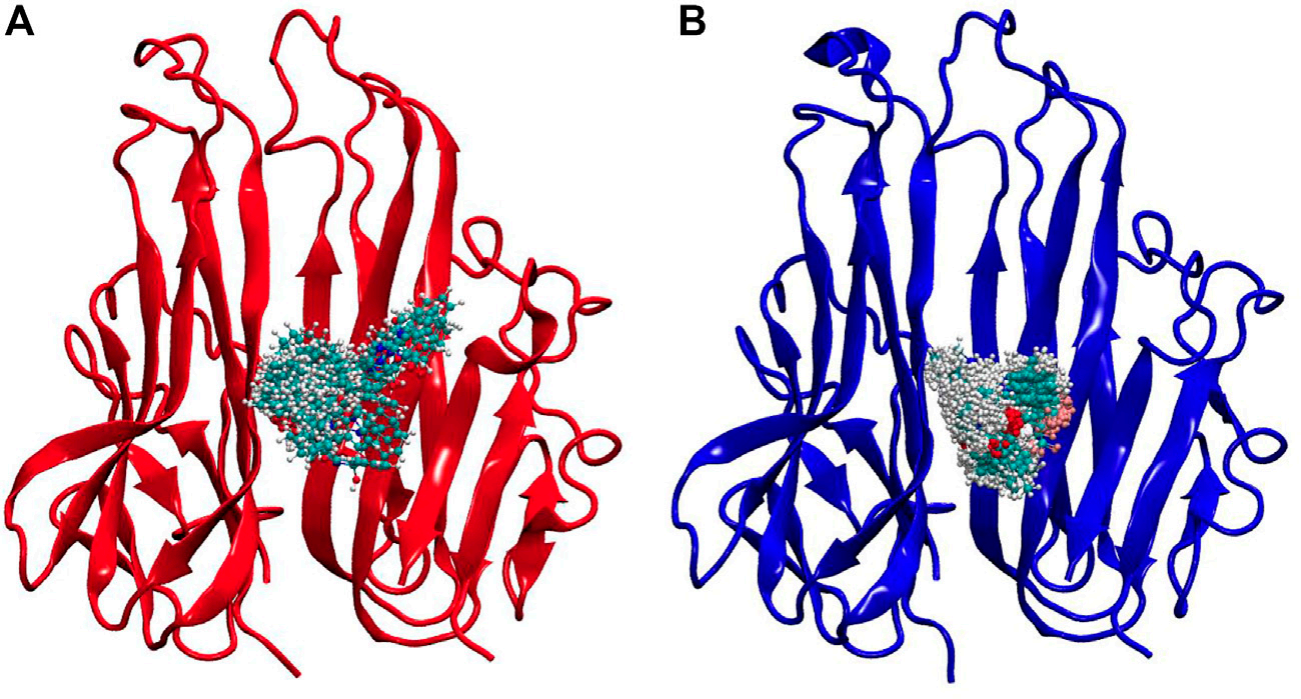
Simulation snapshots of ligands in **(A)** TNF-compound complex and **(B)** TNF-inhibitor complex. The ligand snapshots have been taken at every 1 ns of simulation time, while the protein is fixed at 0 ns (initial structure). The ligand remained in a bound state in both systems, however, **(A)** Is slightly flexible, occupying somewhat more space in the binding site due to increased ligand size, while **(B)** Is highly compact.

Both these ligands gave good binding energy against TNF-α therefore these were further analyzed by molecular dynamic simulation for their distinct properties. This method is unique in its ability to assess protein-ligand systems in aqueous solution at physiological conditions, enhancing the computational model’s resemblance to the actual physical-chemical conditions that are observed in an experiment. Water and ions are known to play a significant role in protein structure and dynamics. Thus, the presence of water and ions at room temperature may be able to correct virtual screening and docking results. Additionally, no other computational method for drug design allows for the analysis of the system dynamics at the molecular level (Qaiser et al., 2020). As seen in Figure 7, the protein was fixed at 0 ns, while the ligand snapshots were generated every 1 ns of simulation time (initial structure). Both the (7a) TNF-plant compound and (7b) TNF- 2az5 ligand systems kept the ligand in a bound state, but (7a) was slightly flexible and took a little more area in the binding site due to the larger ligand, whereas (7b) was quite compact.

**FIGURE 8:**
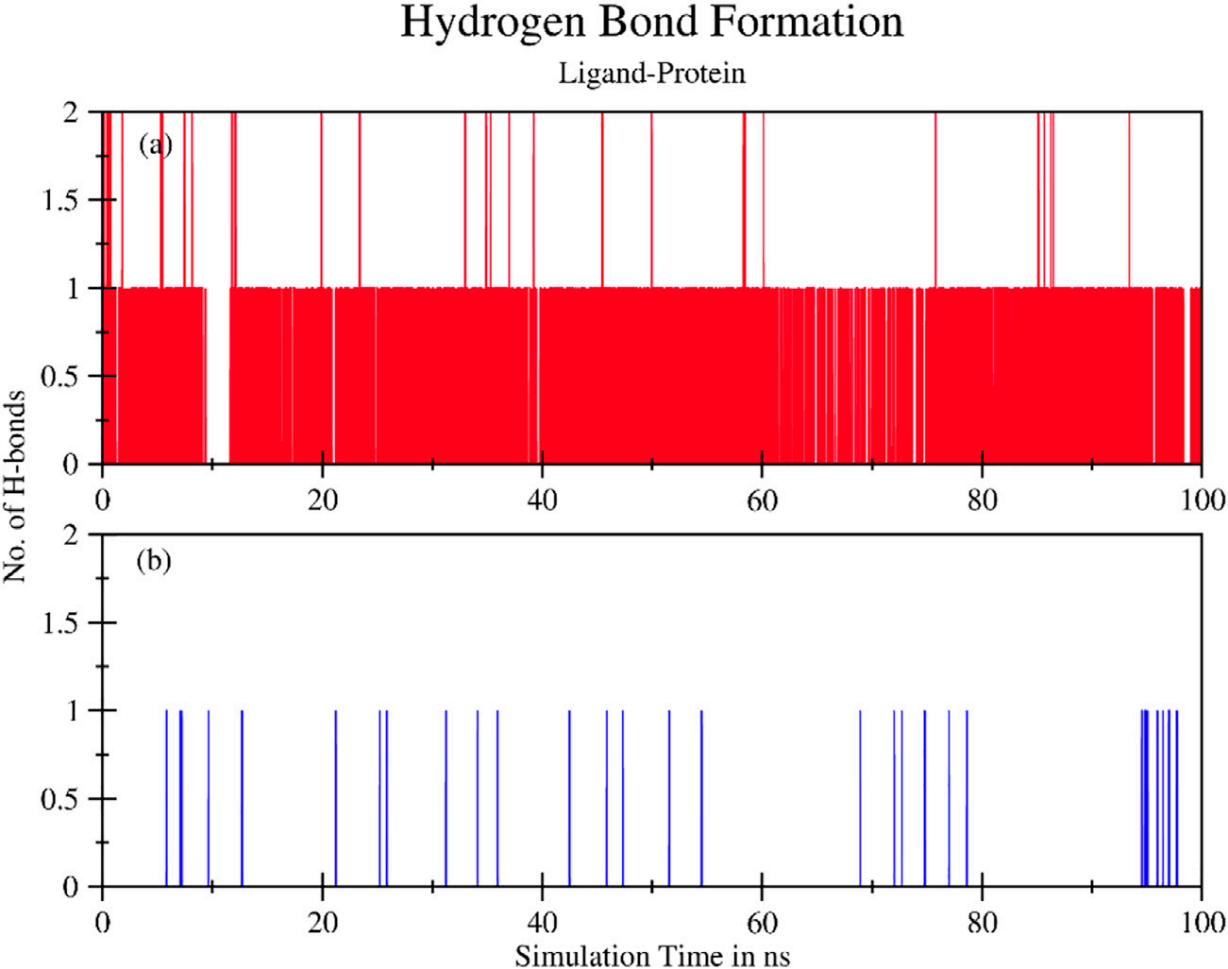
The total number of hydrogen bonds formed between ligand and protein during 100 ns simulation time for **(A)** TNF compound complex and **(B)** TNF (2az5) inhibitor complex. Ligand exhibits hydrogen bonds and forms consistent interactions with protein in the **(A)** System. In **(B)**No significant H-bonds are observed.

Moreover, several hydrogen bonds were also observed during the simulation process as shown in Figure 8. A higher number of hydrogen bonds are an indicator of the strong protein-ligand affinity by end of 100 ns simulation time. Plant ligands exhibited hydrogen bonds and formed consistent interactions with proteins in the TNF-ligand system. However, no significant H-bonds were observed in the standard TNF-2az5 ligand system indicating that plant compound 4-(1-hydroxy-3-oxo-1H-isoindol-2-yl) benzoic acid (PubChemID 18873897) is a suitable candidate molecule for inhibiting TNF-α and derivatives of this compound can be utilized in the management of various other disease pathways.

## 4 Discussion

Rheumatoid arthritis is a chronic inflammatory disease that destroys cartilage and joints. It causes swelling of the synovial lining, leading to immobility. The precise etiology of RA is still unclear, but it is well-known to be caused by genetic and environmental factors. The main aim of the current study was to identify and evaluate the bioactive compounds of *D. viscosa* for their therapeutic potential against rheumatoid arthritis. *D. viscosa* is reported to have anti-inflammatory, antioxidant, anti-microbial, cytotoxic, and wound healing (Malik et al., 2022).

To obtain our objective, several phytochemical tests were performed on the ethanol extract of *D. viscosa*. The results were positive for alkaloids, phenols, flavonoids, tannins, coumarins, terpenoids, sterols, saponins, steroids, and cardiac glycosides. Whereas, negative results were shown for glycosides, anthraquinones, and anthocyanins. After that, total phenolic content (TPC) and total flavonoid content (TFC) both were calculated for the ethanol extract to obtain the quantitative phytochemical analysis which indicated the antioxidant potential of *D. viscosa*. Similar results of the phytochemical analysis were observed in a few other studies (Hossain, 2019; Yeshi et al., 2022).

The antioxidant potential of *D. viscosa* leaves (ethanol extract) was evaluated by two methods including 1) 1, 1-Diphenyl-2-picrylhydrazyl (DPPH) free radical scavenging activity and 2) Ferric Reducing Antioxidant Power (FRAP) assay. The results of DPPH indicated that *D. viscosa* possesses a significantly higher antioxidant potential as compared to standard ascorbic acid with a *p*-value of 0.0018, which is less than 0.05. Related studies have been reported previously which prove its antioxidant potential (Ramkumar and Periyasamy, 2022). The results of the FRAP assay indicated that the ferric-reducing potential of the plant increased with the increasing concentration but was slightly lower as compared to the standard drug. The results were significant with a *p*-value ≤0.0001. The graph trend line demonstrated the extract’s antioxidant activity, which increased with concentration (Malik et al., 2022). Therefore, both assays establish that *D. viscosa* has good antioxidant potential.

The anti-inflammatory potential was evaluated for ethanol extract of *D. viscosa* leaves by two *in vitro* methods including 1) Protein denaturation and 2) Human Red Blood Cell (HRBC) Membrane stabilization assay. The results indicated that plant extract had significantly higher percentage inhibition with a *p*-value of 0.01897 ≤ 0.05, demonstrating that the plant extract was effective in inhibiting the denaturation of protein as reported in previous studies (Khan et al., 2019). Similarly, the results of the HRBC membrane stabilization assay demonstrated that plant extract had a higher percentage inhibition as compared to standard at 620 nm. The results were significant with a *p*-value of 0.00132, which is lower than 0.05. Therefore, the extract of *D. viscosa* plays a vital role in the stabilization of cellular membranes which get affected during the disease prognosis.

Drug development has undergone a remarkable transformation due to *in silico* analysis and bioinformatics techniques. It has cut down the time and money needed to develop new medications. Multiple methods are used in the process of drug discovery. This study applied the Gas chromatography mass-spectrometry (GCMS) technique to obtain bioactive compounds. GCMS gave a list of bioactive compounds present in *D. viscosa* among which the best biocompatible compounds were shortlisted. HUB gene analysis presented TNF-α as the top RA gene among the top 200 RA targets. Molecular docking analysis demonstrated that 4-(1-hydroxy-3-oxo-1H-isoindol-2-yl) benzoic acid (PubChem ID 18873897) showed the best binding energy when docked with TNF-α indicating that this compound could be a potent inhibitor of TNF-α. The results were validated with the re-docking of the TNF (2AZ5) complex. Similarly, the same docking protocol previously reported the inhibitory effect of a flavonoid named viscosine (Khan et al., 2013). Molecular Dynamics simulation is a valuable tool for validating experimentally distinct biological activities by identifying the binding interactions between a protein and its inhibitor (Qaiser et al., 2020). The results of this study indicated that the plant compound (PubChem ID 18873897) can be a potential novel TNF-α inhibitor.

## 5 Conclusion

This study was primarily focused on finding new plant-based therapeutic small molecule inhibitors against Rheumatoid arthritis using *in vitro* and *in silico* techniques. The study concludes that *D. viscosa* and its bioactive compounds have good anti-rheumatic potential. According to *in vitro* findings, the plant extract exhibits exceptional antioxidant and anti-inflammatory properties, which are key components of anti-rheumatic treatments. *In silico* study using computer-aided drug design (CADD) techniques like molecular docking and MD simulation specified the bioactive compounds that have the potential to inhibit rheumatic pathways and show anti-rheumatic properties. In the current study, the plant compound named 4-(1-hydroxy-3-oxo-1H-isoindol-2-yl) benzoic acid (PubChem ID 18873897), showed excellent inhibitory potential against TNF-α, which plays a significant role in the disease progression. However, additional *in-vitro* and *in-vivo* testing is strongly advised to assess the efficacy of this compound in reducing the effects of Rheumatoid arthritis progression.

## Data Availability

The datasets presented in this study can be found in online repositories. The names of the repository/repositories and accession number(s) can be found in the article/supplementary material.

## Abbreviations

TNF, Tumor necrosis factor; RA, Rheumatoid arthritis; DPPH, 2,2-diphenyl-1-picryl-hydrazyl-hydrate; GCMS, Gas chromatography-mass spectrometry; RMSD, Root-mean square deviation; PDB, Protein data bank; DV, Dodonaea viscosa.
